# Phenyl 2‐pyridyl ketoxime induces cellular senescence‐like alterations via nitric oxide production in human diploid fibroblasts

**DOI:** 10.1111/acel.12429

**Published:** 2015-12-22

**Authors:** Kyeong Eun Yang, Hyun‐Jin Jang, In‐Hu Hwang, Young‐Ho Chung, Jong‐Soon Choi, Tae‐Hoon Lee, Yun‐Jo Chung, Min‐Seung Lee, Mi Young Lee, Eui‐Ju Yeo, Ik‐Soon Jang

**Affiliations:** ^1^ Drug & Disease Target GroupDivision of Bioconvergence AnalysisKorea Basic Science InstituteDaejeon305‐333Korea; ^2^ Department of PhysiologyKorea University College of MedicineSeoul02841Korea; ^3^ Department of Oral BiochemistryDental Science Research InstituteChonnam National UniversityGwangju500‐757Korea; ^4^ Center for University‐Wide Research FacilitiesChonbuk National UniversityJeonjuKorea; ^5^ Department of BiochemistryCollege of MedicineGachon UniversityInchon406‐799Korea; ^6^ KM Convergence Research DivisionKorea Institute of Oriental MedicineDaejeon305‐811Korea

**Keywords:** cellular senescence, human diploid fibroblast, nitric oxide, phenyl 2‐pyridyl ketoxime, reactive oxygen species

## Abstract

Phenyl‐2‐pyridyl ketoxime (PPKO) was found to be one of the small molecules enriched in the extracellular matrix of near‐senescent human diploid fibroblasts (HDFs). Treatment of young HDFs with PPKO reduced the viability of young HDFs in a dose‐ and time‐dependent manner and resulted in senescence‐associated β‐galactosidase (SA‐β‐gal) staining and G2/M cell cycle arrest. In addition, the levels of some senescence‐associated proteins, such as phosphorylated ERK1/2, caveolin‐1, p53, p16^ink4a^, and p21^waf1^, were elevated in PPKO‐treated cells. To monitor the effect of PPKO on cell stress responses, reactive oxygen species (ROS) production was examined by flow cytometry. After PPKO treatment, ROS levels transiently increased at 30 min but then returned to baseline at 60 min. The levels of some antioxidant enzymes, such as catalase, peroxiredoxin II and glutathione peroxidase I, were transiently induced by PPKO treatment. SOD II levels increased gradually, whereas the SOD I and III levels were biphasic during the experimental periods after PPKO treatment. Cellular senescence induced by PPKO was suppressed by chemical antioxidants, such as N‐acetylcysteine, 2,2,6,6‐tetramethylpiperidinyloxy, and L‐buthionine‐(*S*,*R*)‐sulfoximine. Furthermore, PPKO increased nitric oxide (NO) production via inducible NO synthase (iNOS) in HDFs. In the presence of NOS inhibitors, such as L‐NG‐nitroarginine methyl ester and L‐NG‐monomethylarginine, PPKO‐induced transient NO production and SA‐β‐gal staining were abrogated. Taken together, these results suggest that PPKO induces cellular senescence in association with transient ROS and NO production and the subsequent induction of senescence‐associated proteins**.**

**Abbreviations**
BSO: L‐buthionine‐(*S*,*R*)‐sulfoximine
BCA: bicinchoninic acid
DCF‐DA: 2′,7′‐dichlorofluorescein diacetate
DMEM: Dulbecco's modified Eagle's medium
ECM: extracellular matrix
ECL: enhanced chemiluminescence
FBS: fetal bovine serum
GPX: glutathione peroxidase
HDFs: human diploid fibroblasts
L‐NAME: L‐NG‐nitroarginine methyl ester
L‐NMMA: L‐NG‐monomethylarginine
MTT: 3‐(4,5‐dimethylthiazol‐e‐yl)‐2,5‐diphenyltetrazolium bromide
NAC: N‐acetylcysteine
NO: nitric oxide
NOS: nitric oxide synthase
PI: propidium iodide
P‐ERK: phosphorylated extracellular signal‐regulated kinase
Prdx: peroxiredoxin
PD: population doubling
PI: propidium iodide
PPKO: phenyl 2‐pyridyl ketoxime
ROS: reactive oxygen species
SA‐β‐gal: senescence‐associated β‐galactosidase
SOD: superoxide dismutase
TEMPO: 2,2,6,6‐tetramethylpiperidinyloxy
X‐gal: 5‐bromo‐4‐chloro‐3‐indolyl‐β‐d‐galactopyranoside

## Introduction

Human diploid fibroblasts (HDFs) undergo terminal growth arrest called cellular senescence. Senescent HDFs differ from young HDFs by a flat and enlarged morphology, enhanced senescence‐associated β‐galactosidase (SA‐β‐gal) activity, resistance to apoptotic insults (Yeo *et al*., 2000a), and hyporesponsiveness to growth factors, such as platelet‐derived growth factor and epidermal growth factor (Yeo & Park, 2002; Yeo *et al*., 2002; Cho *et al*., 2004). It has been reported that cells show growth arrest and senescence‐like phenotypes in response to sublethal concentrations of cytotoxic chemicals, such as mitomycin C, sodium butyrate, doxorubicin, and H_2_O_2_. The molecular changes associated with senescence‐like growth arrest depend on the chemicals used. According to the free‐radical theory of aging (Beckman & Ames, 1998; Haendeler *et al*., 2003; Kurz *et al*., 2004), reactive oxygen species (ROS) are potential candidates for senescence induction, and ROS‐induced oxidative stress may promote cellular senescence. Because ROS are highly reactive molecules, they can damage many cellular components, and thus, they are considered a probable cause of cellular senescence and degenerative aging.

Oximes have the general formula R_1_R_2_C = N‐OH, where R_1_ is an organic side chain and R_2_ may be hydrogen (aldoximes) or another organic group (ketoximes). Oximes are synthesized by condensation between hydroxylamine and aldehydes (in aldoximes) or ketones (in ketoximes). Oximes can also be synthesized by reacting nitrites with compounds containing an acidic hydrogen atom. Ketoximes can be synthesized from natural products, such as terpenoids (Banerjee & Dureja, 2005; Koca *et al*., 2005; Huang *et al*., 2008), and methyl ethyl ketoximes are important industrial chemicals, for example, they are used as antioxidant and antiskinning agents in alkyd paints.

Because they complex with several transition and heavy metal ions, such as Co^2+^, Ni^2+^, Zn^2+^, Pb^2+^, Fe^2+^, Fe^3+^, Cr^3+^, and La^3+^, some oximes, including methyl 2‐pyridyl ketoxime and phenyl 2‐pyridyl ketoxime, have been used for trace metal determinations in biological samples (Soylak *et al*., 2001; Shokrollahi *et al*., 2008). Some oximes are beneficial because of antifungal activities, which suppress disease development in plants including the pea (Banerjee & Dureja, 2010), and antimicrobial effects against *C. albicans* (Koca *et al*., 2005). Interestingly, oxime metal chelates exhibit higher antimicrobial activities than the free ligands (Shokrollahi *et al*., 2008; Koumousi *et al*., 2012). In fact, oxime‐induced insecticidal, miticidal, and nematocidal activities may be due to their complexation with the binuclear Mn^2+^ cluster of arginases and subsequent enzyme inactivations (Moali *et al*., 2000). Oximes can also be useful as antidotes against organophosphorus nerve agents (Kassa, 2002), because they play some roles in reactivation of acetylcholine esterases by attaching to the phosphorus atom of nerve agents to form an oxime phosphonate and release acetylcholinesterase (Kovarik *et al*., 2004, 2007; Taylor *et al*., 2007). Because ketoximes, which freely diffuse across cell membranes, are diacylated and reside in the lipid regions of animal cells, they may have harmful effects in human. It has been reported that high doses of methyl ethyl ketoxime may induce hematopoietic and central nervous system dysfunctions and result in developmental abnormalities (Schulze & Derelanko, 1993; Burka *et al*., 1998; Derelanko *et al*., 2003). Some ketoximes have also been suggested to act as hepatocarcinogens in rats because their oral and intraperitoneal administrations have been reported to increase 8‐hydroxyguanine levels in the liver DNAs and RNAs of male Sprague Dawley and F344 rats (Hussain *et al*., 1990).

Ketoximes are also known to be metabolized *in vivo* in a manner that produces nitric oxide (NO), and the NADPH‐dependent microsomal metabolism by cytochrome P450 has been implicated in the ketoxime‐induced production of NO and ketones in hepatic tissues (Mansuy *et al*., 1995; Jousserandot *et al*., 1998; Caro *et al*., 2001). In addition, NO synthase (NOS)‐catalyzed production of NO from ketoximes has been suggested to occur in nonhepatic tissues (Glover *et al*., 1999).

Previously, it was found that young HDFs acquired senescence‐like phenotypes in the presence of old extracellular matrix (ECM) (Choi *et al*., 2010). To investigate whether ECM components induce senescence‐like alterations in young HDFs, nonpolar small molecules were extracted from young and old ECMs and their identities were characterized by GC/MS analysis. Because preliminary experiments showed that phenyl 2‐pyridyl ketoxime (PPKO) was one of the small molecules enriched in the extracellular matrix (ECM) of only near‐senescent HDFs, we questioned whether PPKO‐induced NO could induce cellular damage and senescence, and whether NOS might be involved in the induction of NO production by PPKO.

Accordingly, we undertook this study to increase our knowledge of senescence induction by investigating the mechanism responsible for ketoxime‐induced cellular senescence.

## Results

### PPKO induced senescence‐like phenotypes and G2/M cell cycle arrest in young HDFs

**Figure 1:**
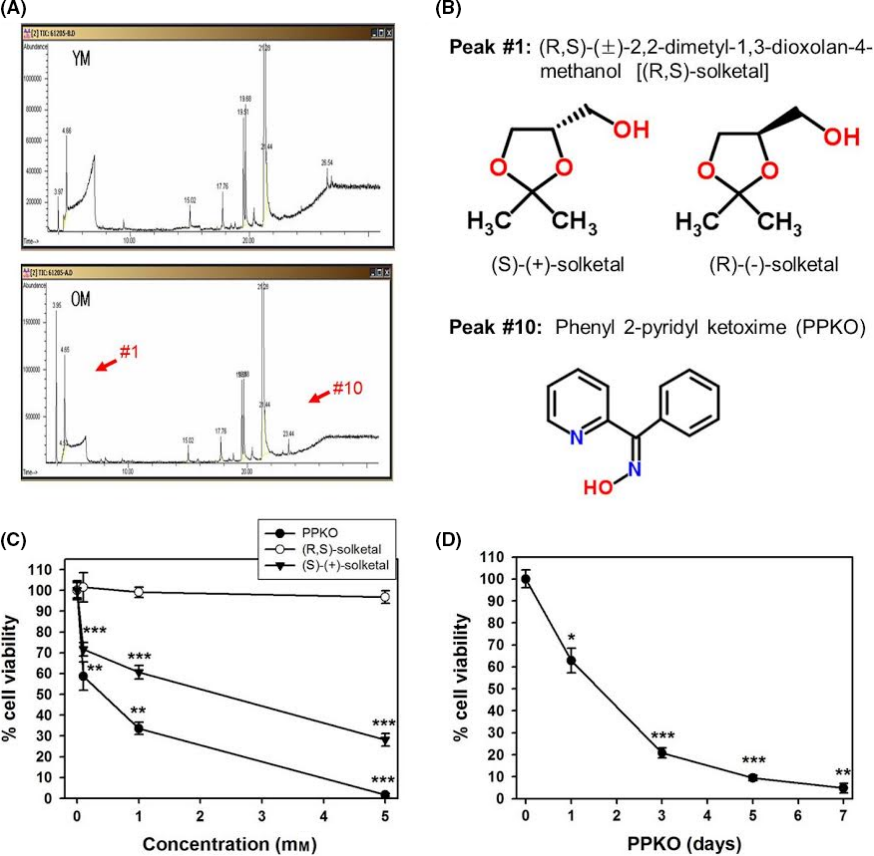
(R,S)‐(±)‐2,2‐dimetyl‐1,3‐dioxolan‐4‐methanol and phenyl 2‐pyridyl ketoxime are small molecules enriched in the ECM of old HDFs. (A) Young and old HDFs were grown for 3 days. Cells were removed and nonpolar small ECM molecules from young and old HDFs were extracted and analyzed by GC/MS. The arrow heads indicate two major peaks that are increased in old ECM compared to young ECM. (B) The structures of (R,S)‐(±)‐2,2‐dimetyl‐1,3‐dioxolan‐4‐methanol [also known as (R,S)‐solketal] and phenyl 2‐pyridyl ketoxime (PPKO) obtained from ChemSpider online chemical database are shown. (C) HDF cells were grown for 1 day and treated with various concentrations (0.5, 1, 5 mM) of (R,S)‐solketal, (S)‐(+)‐solketal, and PPKO for 3 days. (D) HDF cells were grown for 1 day and treated with 1 mM PPKO for the indicated times (1–7 days). Culture media were changed every 2 days. Cell viabilities were determined using MTT assay, and percent viabilities are plotted as the means ± standard deviations of at least three experiments. *P < 0.05, **P < 0.01, and ***P < 0.001 compared with vehicle‐treated control cells.

To determine whether ECM components induce senescence‐like alterations in young HDFs, we extracted and characterized nonpolar small ECM molecules from young and old cells by GC/MS analysis. The identities of all major peaks are listed in Tables 1 and 2. We found two major peaks that were increased in old ECM compared to young ECM (Fig. 1A). The first peak (Peak #1), found at 3.95 of R time, included the racemates of (R,S)‐(±)‐2,2‐dimethyl‐1,3‐dioxolan‐4‐methanol [also known as (R,S)‐solketal]. The second peak, found at 23.44 (peak #10), was characterized as phenyl 2‐pyridyl ketoxime (PPKO). The molecular structures of PPKO and (R,S)‐solketal obtained from ChemSpider online chemical database are shown in Fig. 1B.

**Table 1:** Small molecules derived from the ECM in young HDFs

Peak#	R. Time (min)	Corrected Area	Quality (%)^a^	Name	CAS#
1	3.97	4,113,313	91	(R,S)‐Solketal	100‐79‐8
2	4.66	15,529,445	89	Trimethylethoxysilane	1825‐62‐3
3	15.02	3,712,290	78	Amyl nonanoate	61531‐45‐1
4	17.76	6,417,032	75	Myristamide	638‐58‐4
5	19.51	18,934,505	89	Palmitamide	629‐54‐9
6	19.68	20,850,325	88	Palmitamide	629‐54‐9
7	21.28	400,389,362	95	9‐Octadecenamide	301‐02‐0
8	21.44	15,726,569	89	9‐Octadecenamide	301‐02‐0
9	26.54	3,952,246	–	Hexamethylcyclotrisiloxane^b^	541‐05‐9

a Compounds with quality (%) over 75% were listed.

b Cyclotrisiloxane may be a contaminant derived from column‐coating materials.

**Table 2:** Small molecules derived from the ECM in old HDFs

Peak#	R. Time (min)	Corrected area	Quality (%)^a^	Name	CAS#
1	3.95	25,288,999	93	(R,S)‐Solketal	100‐79‐8
2	4.53	3,904,669	85	Indole	120‐72‐9
3	4.65	34,615,877	88	Trimethylethoxysilane	1825‐62‐3
4	15.02	4,825,192	78	Amyl nonanoate	61531‐45‐1
5	17.76	9,129,173	75	Myristamide	638‐58‐4
6	19.51	22,313,728	91	Palmitamide	629‐54‐9
7	19.68	24,528,236	88	Palmitamide	629‐54‐9
8	21.28	461,093,076	95	9‐Octadecenamide	301‐02‐0
9	21.44	34,063,707	91	9‐Octadecenamide	301‐02‐0
10	23.44	3,867,975	91	Phenyl 2‐Pyridyl Ketoxime	1826‐28‐4

a Compounds with quality (%) over 75% were listed.

We first studied the effect of these compounds on cell viability, monitored by MTT assay. As shown in Fig. 1C, cell viability was greatly reduced by PPKO in a dose‐dependent manner, compared to the (R,S)‐solketal mixture or an enantiomer (S)‐(+)‐solketal. Therefore, we further studied PPKO‐induced reduction of cell viability. As expected, PPKO reduced cell viability in a time‐dependent manner (Fig. 1D). To know the physiological concentrations of PPKO, we compared the corrected peak area for PPKO in old ECM extract to that for standard PPKO with known concentration, analyzed by GC/MS with a SIM quantification mode. Because the concentration of PPKO in old ECM extract was found to be 0.472 mM, we added PPKO to the culture medium at a concentration of 1 mM, more than twice the physiological concentration of PPKO, for the subsequent experiments. At this concentration, treatment with PPKO for 3 days reduced cell viability to 20–30%.

**Figure 2:**
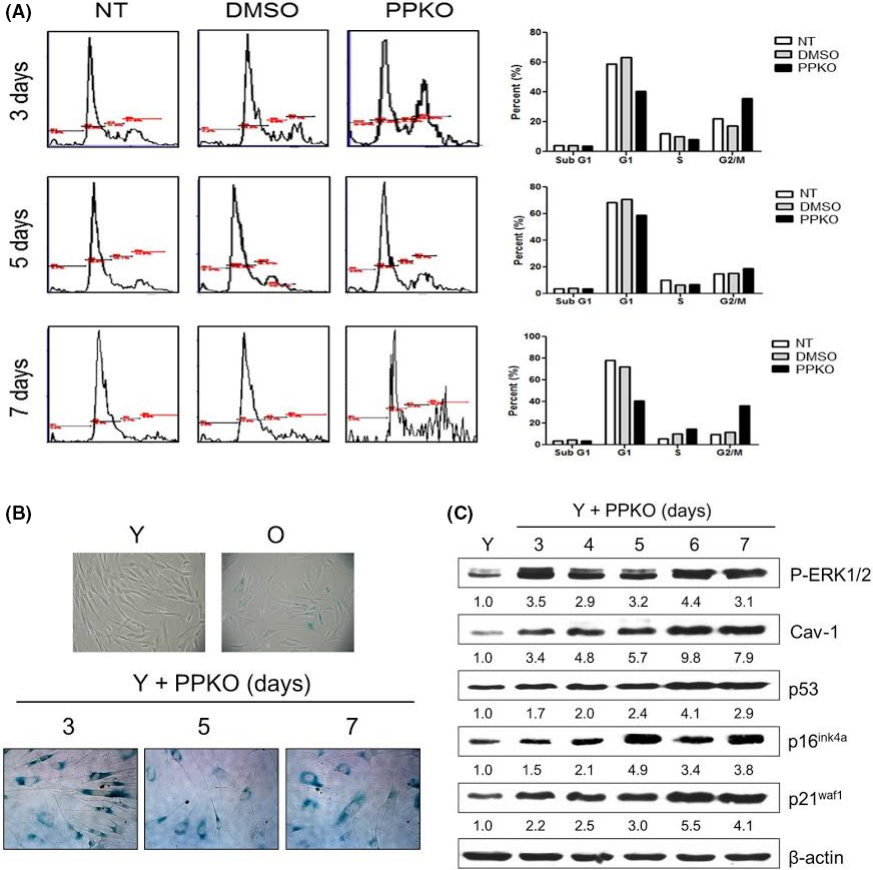
G2/M cell cycle arrest and senescence‐like changes induced by PPKO in young HDFs. A. Cell cycle analysis. B. SA‐β‐gal staining. C. Induction of senescence‐associated proteins in ketoxime‐treated young HDFs. (A) Young HDFs were cultured for 1 day and serum‐depleted by incubation with SFM for 1 day. The cells were then stimulated with 10% FBS in the presence of vehicle or 1 mM PPKO for 3–7 days. The cells were stained with PI, and the cell cycle was analyzed by flow cytometry. The percentage of cells at sub‐G1, Go/G1, S, and G2/M was plotted and shown on the right side. (B) Young HDFs were treated with vehicle (Y) or 1 mM PPKO for the indicated times (3–7 days). Old HDFs (O) were also treated with vehicle for control purposes. Cells were then stained with X‐gal to monitor SA‐β‐gal activities and photographed under an inverted microscope at 100×. (C) Young HDFs were treated with vehicle (Y) or 1 mM PPKO for 3–7 days, and cell lysates were then analyzed by Western blot analysis using antibodies against senescence‐associated proteins (P‐ERK1/2, Cav‐1, p53, p16^ink4a^, and p21^waf1^). β‐actin was used as an internal control. Bands in blots were normalized to β‐actin in each lane. Fold increases vs. levels in lane Y are written under each band.

Reduction in cell viability can be caused by cell cycle arrest, cellular senescence, or cell death. Although the PPKO‐treated cells did not show any visible cell death when treated with 1 mM for 1–7 days, we further investigated whether PPKO causes cell cycle arrest or cell death by flow cytometry after staining the cells with propidium iodide. The cells were serum‐starved for 1 day. After pretreatment for 1 h with vehicle (DMSO) or 1 mM PPKO, or no pretreatment substance, the cells were stimulated with serum (10% FBS) for 3–7 days. The percentages of cells at sub‐G1, G0/G1, S, and G2/M were plotted and are shown on the right side of Fig. 2A. Flow cytometry revealed that there was no significant apoptotic cell death during the experimental periods as judged by unaltered sub‐G1 percentages between DMSO‐ and PPKO‐treated groups at 3–7 days (Fig. 2A). Sorting by DNA content showed that treatment with 1 mM PPKO decreased the number of cells in the G0/G1 fraction from 78.3% to 40.6% but increased the number of cells in the G2/M fraction from 11.4% to 36.2% after 7 days of treatment (Fig. 2A). The data indicate that PPKO can result in G2/M cell cycle arrest.

Because cellular senescence is evidenced by permanent cell cycle arrest as well as elevated SA‐β‐gal expression (Yeo *et al*., 2000b), cellular SA‐β‐gal activity was monitored by X‐gal staining. Treatment of young HDFs with 1 mM PPKO induced morphological enlargement and increased SA‐β‐gal staining in a time‐dependent manner (Fig. 2B), indicating induction of cellular senescence.

Cellular senescence has also been characterized by increases in the levels of some senescence‐associated proteins, such as P‐ERK1/2 (Lim *et al*., 2000), caveolin‐1 (Park *et al*., 2000), and cell cycle regulators like p53, p16^ink4a^, and p21^waf1^ (Yeo *et al*., 2000b). Therefore, to confirm PPKO‐induced cellular senescence, we examined levels of these proteins by Western blot analysis after treating HDFs with 1 mM PPKO for 3–7 days. Bands in blots were normalized to β‐actin in each lane. Fold increases vs. levels in lane Y are written under each band. As shown in Fig. 2C, P‐ERK1/2 levels were increased by 3 days of PPKO treatment and thereafter remained unchanged until day 7. PPKO also gradually increased levels of caveolin‐1, a prime modulator of aging (Cho *et al*., 2004; Cho & Park, 2005) (Fig. 2C), and the levels of the cell cycle regulators p53, p16^ink4a^, and p21^waf1^ (Fig. 2C). The results suggest that PPKO plays a role in induction of cellular senescence.

### PPKO induced the transient accumulation of ROS and the expressions of antioxidant proteins in young HDFs

**Figure 3:**
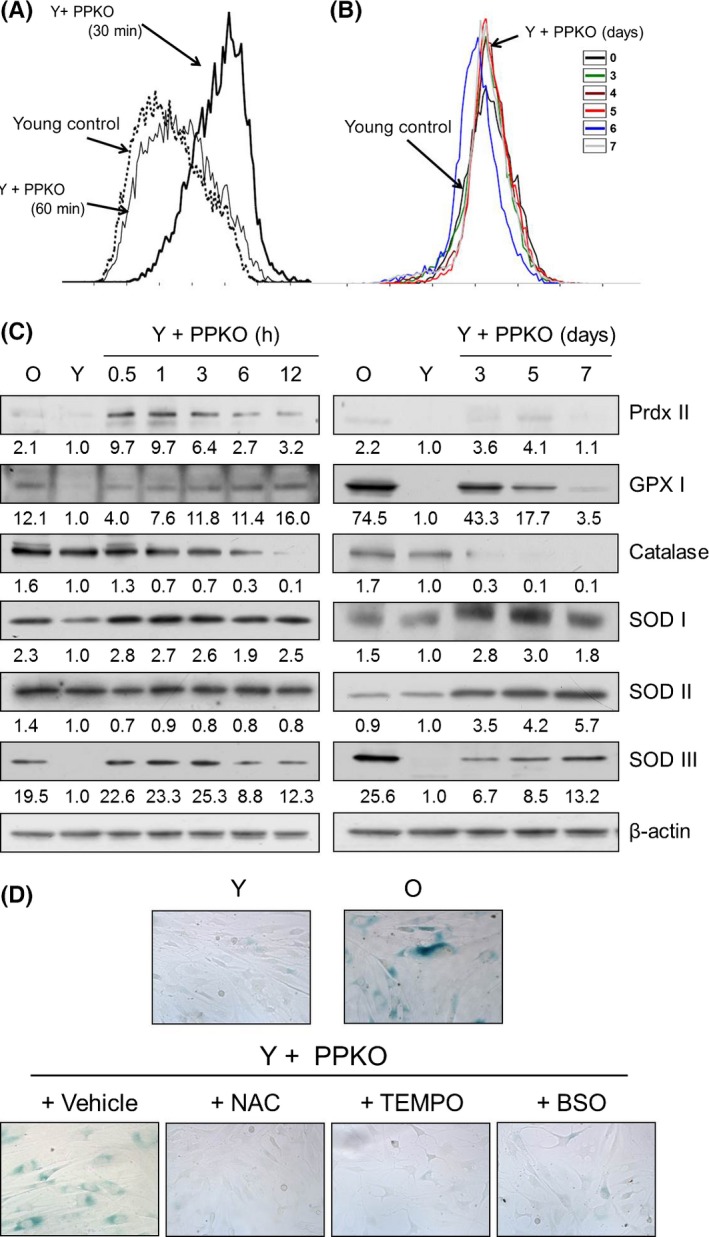
PPKO‐induced ROS generation was responsible for senescence‐like changes in young HDFs. (A and B) Young HDFs from passage 10 were grown for 1 day and treated with 1 mM PPKO for the indicated times (A: short‐term exposure 30 or 60 min, B: long‐term exposure 3–7 days). Cells were then treated with 10 μM DCF‐DA for 30 min. ROS generation was measured by flow cytometry. (C) Young HDFs were treated with vehicle (Y) or 1 mM PPKO for 0.5‐12 h (short‐term) or 3–7 days (long‐term), and cell lysates were separated by SDS‐PAGE. The protein levels of antioxidant enzymes (Prdx II, GPX I, catalase, SOD I, II and III) and β‐actin were assessed by Western blotting. Bands in blots were normalized to β‐actin in each lane. Fold increases vs. levels in lane Y are written under each band. (D) Young HDFs (Y) were pretreated with vehicle or antioxidants (10 mM NAC, 2 mM TEMPO, or 5 mM BSO) for 1 h and then treated with 1 mM PPKO for 3 days. Old HDFs (O) were also treated with vehicle for control purposes. Cells were then stained with X‐gal to monitor SA‐β‐gal activities and photographed under an inverted microscope at 100×.

The involvement of ROS in degenerative senescence has been proposed previously (Beckman & Ames, 1998; Haendeler *et al*., 2003; Kurz *et al*., 2004). To investigate the molecular mechanism for PPKO‐induced cellular senescence, we examined whether PPKO induces the accumulation of ROS in young HDFs. Cells were treated with 1 mM PPKO. After the indicated incubation periods (30 and 60 min, and 3–7 days), cells were treated with 10 μm DCF‐DA at 37°C for 30 min and cellular ROS levels were then measured using flow cytometry. Results showed a PPKO‐induced increase in ROS generation at 30 min (Fig. 3A). Interestingly, ROS levels returned to baseline at 60 min. Long‐term treatment with PPKO did not cause ROS accumulation at 3–7 days (Fig. 3B).

Because we found that PPKO treatment induced transient ROS accumulation, we attempted to determine whether PPKO induces antioxidant enzymes and proteins in young HDFs. After the indicated incubation periods (0.5–12 h and 3–5 days) with 1 mM PPKO, cells were lysed and the levels of antioxidant proteins were assessed by Western blotting. Results revealed the differential expression patterns of antioxidant enzymes (Fig. 3C). A rapid induction at 30 min and then a gradual decrease were observed for Prdx II and catalase. We also observed a gradual increase in GPX I during the early treatment period (0.5–12 h). The GPX I level peaked at day 3 and reduced thereafter. SOD II (mitochondrial SOD) levels remained unchanged during the early period but increased gradually during the later period (3–7 days). However, the expression of SOD I (soluble and cytosolic SOD) and III (extracellular SOD) was biphasic with an early induction and a gradual decrease and then delayed induction at the later period. These observations suggest PPKO induces antioxidant enzymes in different manners and ROS production might be reduced by antioxidant enzymes in HDF cells.

We then investigated the role of transient ROS production in PPKO‐induced cellular senescence in HDFs. Cells were pretreated with well‐known chemical antioxidants, that is, 10 mM NAC, 2 mM TEMPO, or 5 mM BSO for 1 h and then treated with 1 mM PPKO for 3 days. Levels of SA‐β‐gal staining (an indicator of cellular senescence) were compared with those of 0.1% DMSO‐treated young HDFs as control. We found PPKO‐induced SA‐β‐gal staining was reduced by treatment with the antioxidants NAC and TEMPO (Fig. 3D). These results suggest that transient ROS accumulation induced by PPKO induces senescence‐like phenotypes in HDFs.

### PPKO increases NO production by inducing iNOS

**Figure 4:**
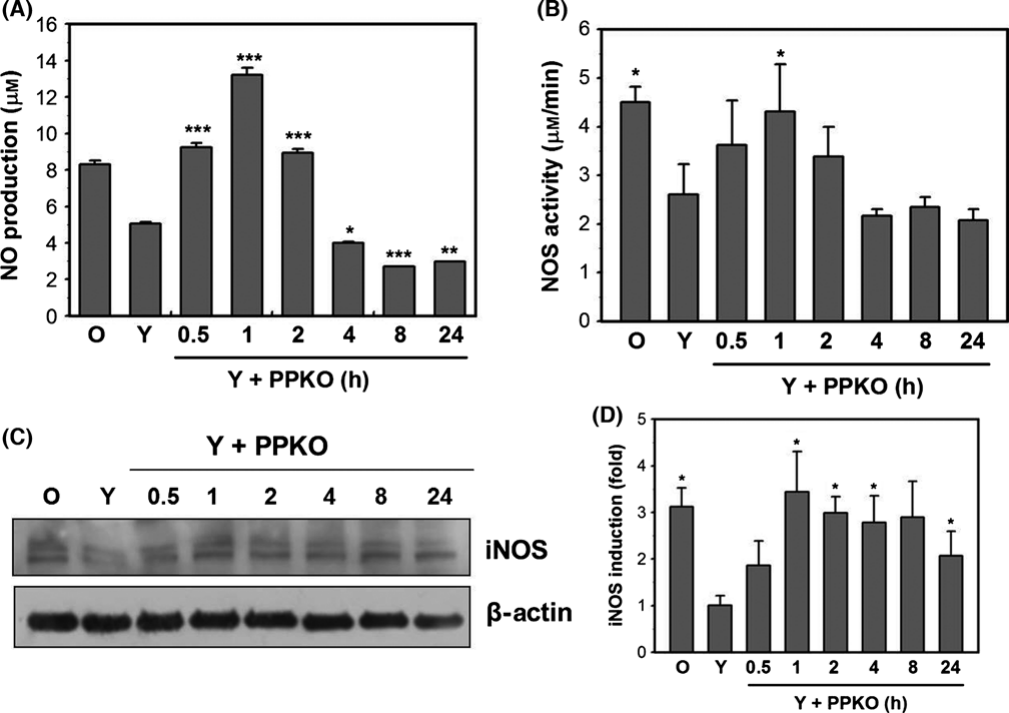
PPKO increased NO levels via iNOS induction. (A) Young HDFs (Y) were grown for 1 day and treated with 1 mM PPKO for the indicated times (0.5–24 h). Old HDFs (O) were treated with vehicle for control purposes. NO concentrations were assayed using an NO detection kit based on the Griess method. (B) Young HDFs were grown for 1 day and treated with 1 mM PPKO for the indicated times (0.5–24 h). NOS activities were determined as described in Materials and Methods. Levels of NO (A) and NOS activities (B) are plotted as the means ± standard deviation of at least three experiments. (C) Young HDFs were treated with vehicle (Y) or 1 mM PPKO (K) for the indicated times (0.5–24 h). Old HDFs were also treated with vehicle for control purposes. Cell lysates were separated by SDS‐PAGE, and the protein levels of iNOS and β‐actin were assessed by Western blotting. Bands in blots were normalized to β‐actin in each lane. Fold increases vs. levels in lane Y were calculated and are plotted as a bar graph (D). *P < 0.05, **P < 0.01, ***P < 0.001 compared with vehicle‐treated young HDFs (Y).

NOS catalyzes the oxidation of acetoxime to produce NO in nonhepatic tissues (Glover *et al*., 1999). Therefore, we considered ketoximes might also be metabolized *in vivo* to NO in a NOS‐dependent manner. Therefore, we monitored NO levels after PPKO treatment using the NO detection kit. Our data showed that 1 mM PPKO induced NO production at around 0.5–2 h post‐PPKO treatment and that NO levels peaked at 1 h (Fig. 4A). We also examined NOS activities using the NO detection kit and NOS protein levels by Western blot analysis. The NOS activity was found to be elevated at ~1 h after PPKO treatment (Fig. 4B), and Western blot analysis showed iNOS protein levels were also increased at this time point (Fig. 4C). Bands in blots were normalized to β‐actin in each lane of Fig. 4C and fold increases vs. levels in lane Y were calculated and are plotted as a bar graph (Fig. 4D). The plot for fold showed a peak at 1 h, and thereafter, the fold was slightly reduced but significant (*P* < 0.05) increase in iNOS expression was sustained at 2–24 h after 1 mM PPKO treatment.

### PPKO‐induced ROS production played a role in NO production and cellular senescence by inducing iNOS protein levels

**Figure 5:**
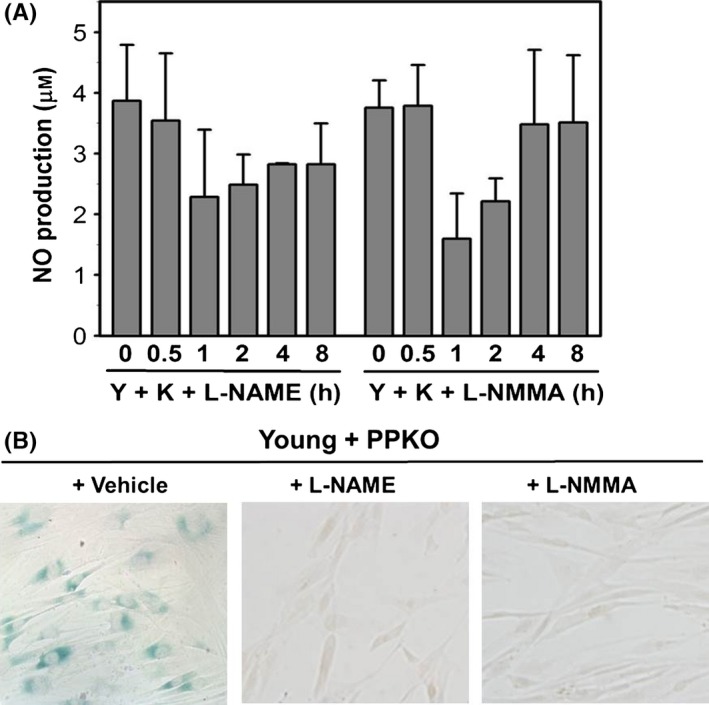
The effect of iNOS inhibitors on PPKO‐induced NO production and SA‐β‐gal staining. (A) Young HDF cells (Y) were grown for 1 day and pretreated with vehicle or iNOS inhibitors (0.5 mM L‐NAME or 0.1 mM L‐NMMA) for 6 h and then treated with 1 mM PPKO (K) for the indicated times (0–8 h). NO concentrations were determined using an NO detection kit as described in Materials and Methods. (B) Young HDFs (Y) were pretreated with vehicle or iNOS inhibitors (L‐NAME or L‐NMMA) for 6 h and then treated with 1 mM PPKO (K) for 3 days. Cells were then stained with X‐gal to monitor SA‐β‐gal activities, and the stained cells were photographed under an inverted microscope at 100×.

The role of iNOS in PPKO‐induced NO production was confirmed by pretreating HDFs with either of two iNOS inhibitors L‐NAME (0.5 mM) and L‐NMMA (0.1 mM) for 6 h, and then treating cells with 1 mM PPKO for the indicated times. We found that both iNOS inhibitors abrogated PPKO‐dependent NO production in HDFs (Fig. 5A). In addition, the role of iNOS‐dependent NO production in PPKO‐induced cellular senescence was also investigated by SA‐β‐gal staining in HDFs after pretreating cells with these iNOS inhibitors and then treating with PPKO. As shown in Fig. 5B, PPKO‐induced SA‐β‐gal staining was abrogated by pretreatment with the iNOS inhibitors. These results suggest that iNOS‐dependent NO production is involved in PPKO‐induced cellular senescence in HDFs.

**Figure 6:**
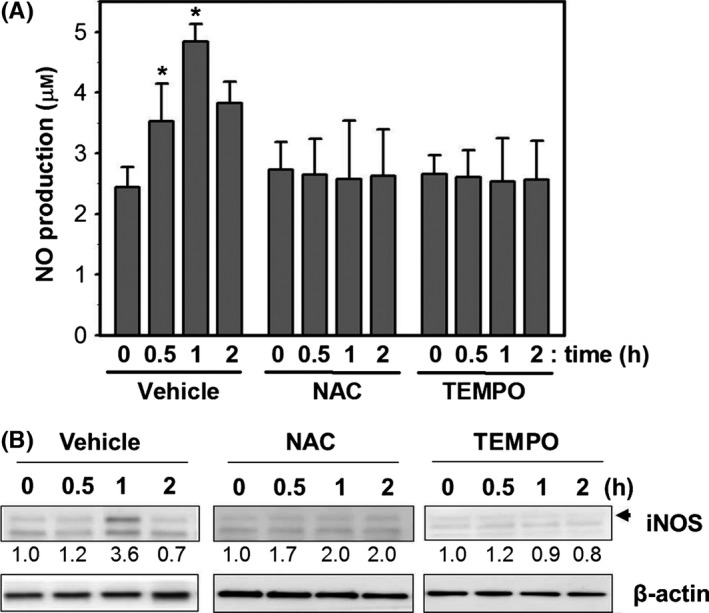
PPKO increased NO production via ROS‐dependent iNOS induction. (A) Young HDF cells (Y) were grown for 1 day, pretreated with vehicle or antioxidants (10 mM NAC or 2 mM TEMPO) for 1 h, and then treated with 1 mM PPKO (K) for the indicated times (0–2 h). NO concentrations were determined using an NO detection kit as described in Materials and Methods. (B) Young HDFs (Y) were pretreated with vehicle or antioxidants (10 mM NAC or 2 mM TEMPO) for 1 h and then treated with 1 mM PPKO (K) for the indicated times (1–2 h). Cell lysates were separated by SDS‐PAGE, and protein levels of iNOS and β‐actin were assessed by Western blotting. Bands in blots were normalized to β‐actin in each lane. Fold increases vs. levels in 0 h are written under each band. *P < 0.05 compared with vehicle‐pretreated young HDFs at 0 h after PPKO treatment.

To investigate whether PPKO‐induced transient ROS accumulation plays a role in iNOS induction and subsequent NO production, cells were pretreated with vehicle, NAC, or TEMPO for 1 h and then treated with vehicle or 1 mM PPKO for 1 h. NO production was measured using the NO detection kit and iNOS protein expression by Western blot analysis. The results obtained showed that pretreatment with either antioxidant significantly reduced PPKO‐induced NO production (Fig. 6A) and iNOS protein expression (Fig. 6B). Taken together, these results indicate PPKO‐induced early ROS production causes the induction of iNOS protein and subsequent NO production, and suggest that PPKO‐induced transient NO production plays a role in senescence‐like alterations in PPKO‐treated HDF cells.

## Discussion

The present study shows that phenyl 2‐pyridyl ketoxime (PPKO; a small molecule enriched in old ECM) induces senescence‐like phenotypes, such as diminished cell viability, SA‐β‐gal staining, and elevated levels of senescence‐associated proteins, in young HDFs (Fig. 2). Increases in the levels of Cav‐1 and CDK inhibitors, such as p53, p16^ink4a^, and p21^waf1^, have been implicated in the aging process. As shown in Fig. 2C, the levels of these proteins after 3 day treatment with ketoxime were higher than in the vehicle‐treated young HDFs (3.4‐, 1.7‐, 1.5‐, 2.2‐fold increases, respectively) and these levels gradually increased during days 5–7. While the cell cycle was arrested at G2/M in some cells and cellular senescence was obvious at day 3 after PPKO treatment, the cellular contents responsible for senescence might further increase to result in fully senescent cells. We also observed this phenomenon in other systems. The levels of these proteins increased proportionately with the increase in the passage of culture, and hydroxyurea‐induced premature senescence also induced gradual increases in these proteins (Yeo *et al*., 2000b).

The free‐radical theory of aging posits that degenerative senescence is largely the result of the cumulative effect of ROS (Haendeler *et al*., 2003; Kurz *et al*., 2004), and the present study demonstrates that PPKO induced ROS generation in 30 min (Fig. 3) and that the antioxidants NAC and TEMPO abrogated PPKO‐induced SA‐β‐gal staining in young HDFs (Fig. 3D). These observations indicate that PPKO‐induced ROS accumulation *per se* might cause oxidative stress and senescence‐like changes in PPKO‐treated young HDFs. However, PPKO‐induced ROS generation was transient in nature, that is ROS accumulated after 30 min of PPKO treatment and then returned to baseline (Fig. 3A and B). We also observed NO production at around 1 h post‐PPKO, but NO levels also returned to baseline at 4–24 h (Fig. 4A). It is not known whether the transient accumulations of ROS and NO play a critical role in inducing the senescence‐like phenotypes observed in PPKO‐treated HDF cells. Because endogenously generated NO has also been suggested to act as a ROS scavenger, and to protect exponentially growing cells from ROS‐induced apoptosis (Wartenberg *et al*., 2003), it is possible that NO to ROS ratio may determine cellular fates with respect to apoptosis or senescence.

Previously, an oxime (3‐(phenylhydrazono) butan‐2‐one oxime) was found to act as a potential antioxidant *in vitro* and in mice (Puntel *et al*., 2008). *In vitro,* hydrogen peroxide‐induced and malonate‐iron mixture‐induced lipid peroxidations were decreased by low concentrations of this oxime (0.1–1.0 μm). However, because basal lipid peroxidation levels were not altered, low doses of this oxime may not act as a prooxidant or antioxidant. If so, additional factors might be involved in oxime‐induced oxidative stress amelioration. In contrast, a high dose of methyl ethyl ketoxime might be cytotoxic and induce hematopoietic and central nervous system dysfunctions, and thus developmental abnormalities (Schulze & Derelanko, 1993; Burka *et al*., 1998; Derelanko *et al*., 2003). In the present study, we also observed that PPKO at 1 mM increased ROS (Fig. 3) and NO (Fig. 4A) levels and that this resulted in senescence‐like alterations in HDFs (Fig. 2). The above‐mentioned results suggest that the physiological effects of PPKO may be dose dependent and cell type specific.

It is now questioned how PPKO induces the production of ROS. The microsomal metabolism of ketoxime is believed to proceed via a transient, cytochrome P450‐catalyzed conversion of their >C = N‐ functional group to a peroxide (Jousserandot *et al*., 1998). In addition, it has been proposed that the nitric oxide synthase (NOS)‐dependent oxidation of ketoximes proceeds via initial formation of an iminoxyl radical (Korth *et al*., 1994). Caro *et al*. (Caro *et al*., 2001) suggested that ROS play a key role in the oxidation of acetoxime to NO by liver microsomes through a mechanism involving H abstraction from the OH moiety by hydroxyl radical‐like oxidants in a NOS‐independent manner. Although it is not clear which mechanism is involved in our cell system, the ketoxime‐derived peroxide and radicals might be responsible for transient ROS production. The ketoxime radical can be subsequently converted to NO and ketone in either case.

The increase in oxidative potential and the loss of proper antioxidant defense appear to be involved in the aging process. The differential changes in antioxidant enzymes, such as catalase and SODs, have been reported in many aging systems. Their expression and activity patterns were similar during the aging process in certain systems, but not coincident in other systems. It was reported that catalase activity in the rat brain increases with age and notably peaks at 12 months but decreases thereafter until 21 months (Tsay *et al*., 2000). The total SOD and Mn SOD activities show a similar trend and exhibit higher levels at 6–12 months. Other reports show that older cells and tissues possess ineffective catalase activity so that they cannot counteract ROS‐induced damage (Conti *et al*., 2015; Griffith *et al*., 2015). We observed that ketoxime caused a rapid induction of catalase at 30 min followed by a gradual decrease. Therefore, we postulate that decreased catalase activity might play a partial role in senescence induction by ketoxime.

In certain systems, catalase activity decreases but SOD activity increases in aged cells and tissues, we observed a similar phenomenon in ketoxime‐induced aging. Ketoxime caused a rapid induction and then a gradual decrease in catalase and other antioxidant enzymes, such as Prdx II. Interestingly, SOD II (mitochondrial SOD) levels increased gradually during the later periods (3–7 days), whereas the SOD I (soluble and cytosolic SOD) and III (extracellular SOD) levels were biphasic, with an early induction and gradual decrease thereafter, and then delayed induction at the later time points. SOD and catalase are known to be two prime antioxidant enzymes. Increased levels of antioxidant enzymes were predicted to reduce oxidative stress and extend lifespan. Transgenic fruit flies that produce either more SOD or more catalase alone do not live longer than the average fruit fly, but transgenic fruit flies that simultaneously overexpress both enzymes have increased average lifespans, jumping from 45 to 75 days. A likely explanation is that SOD and catalase collaborate to remove free radicals: SOD converts the O_2_ to H_2_O_2_; catalase, in turn, converts H_2_O_2_ to molecular oxygen (O_2_) and water. Increasing SOD levels without simultaneously increasing catalase levels would increase the concentration of H_2_O_2_, a reactive oxygen species that can cause damage. At this moment, we do not know why ketoxime causes a rapid induction and then a gradual decrease in catalase, Prdx II, and SOD I, or why the expression patterns of SOD II and SOD III are different from that of SOD I. It remains to be clarified by further studies.

ROS lead to activation of transcription factor nuclear factor‐kappa B (NF‐*k*B), which is responsible for the activation of the iNOS promoter (Bubici *et al*., 2006). Therefore, reduction of ROS should reduce iNOS expression. Induction of the high‐output iNOS usually occurs in an oxidative environment, and thus, high levels of NO have the opportunity to react with superoxide to affect cell toxicity. These properties may define the roles of iNOS in host immunity, enabling its participation in antimicrobial and antitumor activities as part of the macrophage oxidative burst (Mungrue *et al*., 2002). Ketoxime treatment induces transient ROS production which might cause transient iNOS induction, resulting in NO production. NO can play a negative feedback regulatory role in NOS activity. Although iNOS is less sensitive than other NOSs to inhibition by NO, NO generation by iNOS in relatively large quantities may lead to a negative feedback effect. The mechanism by which NO inhibits NOS involves the heme iron prosthetic group and nitrosylation of NOS (Griscavage *et al*., 1995).

Interactions between NO and ROS in cellular redox signaling are important determinants of several physiological responses. For example, the activation of NRF2, leading to induction of antioxidant defense genes, is stimulated by NO through nitrosylation of KEAP1 (McMahon *et al*., 2010), but is also regulated by ROS (Yamamoto *et al*., 2008) and other oxidatively modified molecules (Ishii *et al*., 2004). ROS can modify reactive cysteine residues of KEAP1 to increase NRF2 activity (Yamamoto *et al*., 2008). The balance among NO, ROS, and antioxidant enzyme expression via NRF2 may determine the consequence of oxidative stress induction.

The roles of NO and its by‐products, such as nitrate (${\text{NO}}_{3}^{-}$), nitrite (${\text{NO}}_{2}^{-}$), peroxynitrite (ONOO^‐^), and 3‐nitrotyrosine, have been shown to be extremely diverse in mammals, and to range from blood pressure regulation to neurotransmission, cellular signaling, vasodilation, immune response, host defense, and aging (Drew & Leeuwenburgh, 2002). Ketoximes have been shown to produce NO *in vivo* via NADPH‐dependent microsomal metabolism by P450 in hepatic tissues (Mansuy *et al*., 1995; Jousserandot *et al*., 1998; Caro *et al*., 2001). Furthermore, acetoxime was not found to be active as a substrate or as an inhibitor of iNOS in E47 HepG2 hepatic cell line, which expresses CYP2E1, and thus, it was suggested that acetoxime‐dependent NO generation can be catalyzed by cytochrome P450 but not by NOS in hepatic cell lines (Caro *et al*., 2001). In contrast, NOS was shown to metabolize ketoxime to produce NO in nonhepatic tissues (Glover *et al*., 1999). Because HDF cells are nonhepatic cells, it has been postulated that NOS is involved in NO production after PPKO treatment. Our data show that PPKO induced NO production transiently at around 1 h post‐treatment (Fig. 4A) and that this occurred concurrently with an increase in iNOS protein levels (Fig. 4C). Because the iNOS inhibitors, L‐NAME and L‐NMMA, abrogated NO production (Fig. 5A) and SA‐β‐gal staining (Fig. 5B), we suggest that iNOS‐dependent NO production is a factor of PPKO‐induced senescence‐like alterations in HDFs.

The present study shows that PPKO induced transient ROS production at 30 min (Fig. 3A) and the protein levels of antioxidant enzymes, such as Prdx II, GPX I, catalase, and SOD I, II, and III in HDF cells (Fig. 3C). These results suggest antioxidant enzymes might reduce ROS levels; therefore, ROS productions are transient. Previously, Fenton‐like system (Fe^2+^ plus H_2_O_2_) and hydroxyl radical‐like oxidants were proposed to be involved in the oxidation of acetoxime to NO in the E47 HepG2 hepatic cell line (Caro *et al*., 2001), in which antioxidant enzymes, such as SOD and catalase, significantly inhibit microsomal NO production from acetoxime (Caro *et al*., 2001). Similarly, in the present study, we observed the antioxidants NAC and TEMPO reduced PPKO‐induced NO production (Fig. 6A), iNOS induction (Fig. 6B), and SA‐β‐gal staining (Fig. 3D). It was suggested that an increase in ROS upregulates NOS expression *in vitro* in human coronary artery endothelial cells grown in culture and *in vivo* in animals via NF‐ϰB activation (Zhen *et al*., 2008). It has also been shown that ROS production induced by GSH depletion, caused by combination of BSO plus HCH treatment, increased the production of NO by iNOS by activating the JNK‐mediated ERK pathway and led to the apoptosis of chronic myeloid leukemic cells (Chowdhury *et al*., 2013). Although the molecular mechanism responsible for iNOS induction by ROS is not known in HDFs, PPKO‐induced ROS production might cause iNOS induction and subsequent NO production.

Taken together, our results suggest that PPKO‐induced ROS production plays a role in subsequent NO production via iNOS induction and that antioxidant enzymes might reduce ROS‐dependent NO production in HDFs.

## Materials and methods

### Materials

The following were reagents that were used in the present study. Dulbecco's modified Eagle's medium (DMEM), 3‐(4,5‐dimethylthiazol‐e‐yl)‐2,5‐diphenyltetrazolium bromide (MTT), trypan blue, N‐acetylcysteine (NAC), 5‐bromo‐4‐chloro‐3‐indolyl‐β‐d‐galactopyranoside (X‐gal), L‐NG‐nitroarginine methyl ester (L‐NAME), 2,2,6,6‐tetramethylpiperidinyloxy (TEMPO), propidium iodide, (R,S)‐solketal, (S)‐solketal, and L‐buthionine‐(*S*,*R*)‐sulfoximine (BSO) were purchased from Sigma‐Aldrich (St. Louis, MO, USA); fetal bovine serum (FBS), penicillin, streptomycin, and other cell culture products were obtained from Life Technologies (Grand Island, NY, USA); L‐NG‐monomethylarginine (L‐NMMA) was from Calbiochem (LaJolla, CA, USA); polyclonal antibodies against phosphorylated extracellular signal‐regulated kinase 1/2 (P‐ERK1/2), caveolin‐1, peroxiredoxin II (Prdx II), glutathione peroxidase I (GPX I), catalase, superoxide dismutases (SOD I, SOD II, and SOD III), p53, p16_ink4a_, and p21_waf1_ were from Cell Signaling Technology (Beverly, MA, USA), and polyclonal antibodies against β‐actin and secondary horseradish peroxidase‐conjugated anti‐rabbit and anti‐mouse antibodies were from Santa Cruz Biotechnology (Santa Cruz, CA, USA); nitrocellulose membranes were purchased from the Pall Corporation (Pensacola, FL, USA); bicinchoninic acid (BCA) protein assay kits and the enhanced chemiluminescence (ECL) system were from Pierce Biotechnology (Lockford, IL, USA); 2′,7′‐dichlorofluorescein diacetate (DCF‐DA) was from Molecular Probes (Eugene, OR, USA); and NO detection kits were purchased from Intron (Seoul, Korea).

### Cell culture

Primary human fibroblasts were isolated from newborn foreskins, as previously described (Boyce & Ham, 1983). Cells were maintained in DMEM containing 10% FBS and antibiotics. The molecular contents of young cells from the early stage of culture (population doubling (PD) ≤25) were compared with those of PD 65–70 senescent cells. Senescent cells were characterized by morphological changes, enhanced SA‐β‐gal activity, and a reduced rate of proliferation (Yeo *et al*., 2000a). Prior to PPKO treatment, cells were grown for 1–2 days to 60–70% confluence in DMEM‐based culture medium.

### Gas chromatography/mass spectrometry of small ECM molecules

Young and senescent HDFs were cultured in 10‐cm culture dishes at 37°C for 3 days and removed by trypsinization with 1 mL of 1 × trypsin‐EDTA for 2 min. Dishes containing ECM were washed twice with ice‐cold PBS and then kept at 4°C for 1 day. ECM was then transferred to Eppendorf tubes, and small molecules were extracted using 100 μL of n‐hexane. To identify nonpolar small metabolites in ECMs, gas chromatography/mass spectrometry (GC/MS) was carried out as described by Hong *et al*. (2003). Briefly, samples dissolved in n‐hexane were applied to an Agilent 6890 GC coupled to an Agilent 5973N mass spectrometer (Palo Alto, CA, USA) equipped with a DB‐5MS silica capillary column (Agilent 6890N, Palo Alto, CA, USA) of dimensions 60 m × 0.25 mm × 0.25 μm (length × i.d. × thickness) coated with chemically bonded 5% phenyl‐methylsilicone. The column was programmed to hold a temperature of 70°C for 3 min and then to increase temperature at 10°C per min to 300°C, which was held for 5 min. The injector temperature was 280°C, the carrier gas was helium (99.999%) at 1.0 mL min^−1^, and inlet head pressure was 12 psi. Samples (1 μL) were injected in split mode (30:1) using 1‐min purge time. Electron energy and ion source temperature were set at 70 eV and 280°C, respectively, and spectra were obtained in the range 50–500 m z^−1^. The peaks were identified by comparing them with a chemical library maintained by National Institute of Standards and Technology (Wiley 275 L).

### MTT assay

Cells were seeded into 12‐well plates at a density of 2 × 10^5^ cells well^−1^ in 1 mL of DMEM‐based culture medium in triplicate, grown for 24 h, and treated with 1 mM of PPKO. After the indicated incubation times at 37°C, 30 μL of MTT solution (5 mg mL^−1^ stock) was added to cells, and incubation continued for 1 h at 37°C. Media were removed carefully, and 300 μL of DMSO was then added to dissolve the blue formazan crystals produced in living cells. Absorbance was read at 540 nm using an ELISA reader (Multiskan EX; Thermo Lab systems, Beverly, MA, USA).

### Western blot analysis

For Western blot analysis, young HDF cells (3 × 10^5^ cells well^−1^) were seeded in 3 mL of medium, grown for 24 h in 6‐cm culture dish, and treated with 1 mM PPKO for the indicated times. HDFs were washed twice with ice‐cold PBS and total cell lysates were prepared in an ice‐cold lysis buffer containing 25 mM Hepes (pH 8.0), 150 mM NaCl, 1 mM EDTA, 1 mM Na_3_VO_4_, 1 mM NaF, 1% Triton X‐100, and protease inhibitor cocktail for 30 min. Whole cell lysates were centrifuged at 12 000 *g* for 10 min at 4°C to remove cellular debris. The protein concentrations of lysates were determined using a BCA protein assay kit. Cell lysates containing equal amounts of protein (45 μg) were resolved by 8–12% SDS–polyacrylamide gel electrophoresis and transferred onto Protran nitrocellulose filters. Blots were blocked with a solution containing 5% nonfat dried milk and 0.1% Tween‐20, treated with antibodies in the blocking solution overnight, washed, and incubated with horseradish peroxidase‐conjugated anti‐rabbit or mouse IgGs (1:5000) in blocking solution for 1 h at RT. Immune complexes were visualized using an ECL system.

### SA‐β‐gal staining

Young and senescent HDF cells were fixed and stained for SA‐β‐gal activity as previously described (Fenton *et al*., 2001). Briefly, cells were washed twice with PBS, fixed with 2% formaldehyde/0.2% glutaraldehyde in PBS for 10 min, washed twice with PBS, and incubated with fresh SA‐β‐gal staining solution containing 1 mg ml^−1^ X‐gal, 5 mM potassium ferrocyanide, 5 mM potassium ferricyanide, and 2 mM MgCl_2_ in 40 mM citric acid/sodium phosphate buffer (pH 6.0) for 12 h at 37°C in a CO_2_ free atmosphere. The SA‐β‐gal‐stained cells obtained were photographed under an inverted microscope (Nikon Eclipse TS100, Nikon, Melville, NY, USA 100×).

### Cell cycle analysis

The effect of PPKO treatment on cell cycle progression was determined by flow cytometry as described previously (Kwon *et al*., 2013). The DNA content was assessed by staining the ethanol‐fixed cells with PI. HDFs (1 × 10^6^ cells mL^−1^) were seeded in 60‐mm tissue culture dishes, and allowed to attach for 1 day. The medium was replaced with fresh medium containing vehicle (DMSO) or 1 mM PPKO. After incubation for the indicated times at 37°C, the cells were harvested, washed twice with PBS, and fixed in ice‐cold 70% ethanol overnight at 4°C. The fixed cells were kept at 4°C and stained immediately before analysis. For staining, the fixed cells were pelleted by centrifugation at 1000 *g* for 5 min and washed twice with PBS containing 0.1% BSA, then incubated with 1 mg mL^−1^ of DNase‐free RNase A and 50 μg mL^−1^ of PI for 1 h at 37°C in the dark. The cells were analyzed using a FACSCalibur flow cytometer (Becton Dickinson, San Jose, CA, USA).

### Measurement of ROS

Intracellular ROS production was assessed based on the formation of a fluorescent compound from DCF‐DA (Ding *et al*., 2004; Hayashi *et al*., 2005). Briefly, HDFs were incubated with 1 mM PPKO for the indicated times, incubated with 10 μm DCF‐DA at 37°C for 30 min, washed twice with ice‐cold PBS, trypsinized, and washed once by centrifugation at 1000 *g* for 5 min to remove trypsin and extracellular PPKO. They were then resuspended in PBS and transferred into 5‐ml round‐bottomed polystyrene tubes with cell‐strainer caps. Tubes were protected from light and stored at 4°C until ready for analysis, which was conducted using a FACS ARIA flow cytometer fusion cell sorter (Becton Dickinson, Franklin Lakes, NJ, USA) at excitation and emission wavelengths of 488 and 525 nm, respectively, using a 30‐nm bandpass. Ten thousand events were analyzed per sample.

### Measurement of NO levels and iNOS activity

NO levels were determined colorimetrically using an NO detection kit based on the Griess method, which indirectly measures NO concentration by determining nitite (${\text{NO}}_{2}^{-}$) concentrations. For the assay, 100 μL of samples was collected and added to wells in triplicate. After adding 50 μL of N1 buffer, the prereaction was allowed to continue for 10 min at RT. N2 buffer (50 μL) was then added and the final reaction was carried out for 10 min at RT. Absorbance between 520 and 560 nm was measured using an ELISA reader (Multiskan EX; Thermo Lab systems). Nitrite levels were determined using a standard curve. Because NOS produces a radical compound NO, during the transformation of L‐arginine to citrulline, the cellular NOS activity in 100 μL cell extract was also determined using the NO detection kit.

### Statistical analysis

All results are presented as the means ± standard deviations of at least three independent experiments. Statistical significance was determined using Student's t‐test using Graphpad Prism, version 5.0 (Graphpad Software Inc, San Diego, CA, USA). *P* values of ≤0.05 were considered statistically significant.

## Funding

No funding information provided.

## Conflict of interest

The authors have no potential conflict of interest to declare.

## Author contributions

The authors have made the following declarations about their contributions: Kyeong Eun Yang and Hyun‐Jin Jang performed the experiments including MTT and Western blotting, NO detection, and they analyzed the data; In‐Hu Hwanf, Young‐Ho Chung, Tae‐Hoon Lee, Jong‐Soon Choi, Yun‐Jo Chung, and Min‐Seung Lee supported their experiments and created the figures; Eui‐Ju Yeo and Ik‐Soon Jang conceived and designed the experiments, contributed reagents, supplied materials, provided analysis tools, and wrote the manuscript.
